# Invited comment on Pescatori: Prevention of postoperative fecal incontinence after anal fistula surgery

**DOI:** 10.1007/s10151-025-03269-2

**Published:** 2026-01-29

**Authors:** M. Pescatori

**Affiliations:** Clinica Parioli, Rome, Italy

## Introduction

John Goligher wrote that it is more difficult to successfully operate on a patient with a complex recurrent anal fistula than on a patient with rectal cancer. Similarly, Steve Wexner wrote that surgery of anal fistulas is the most demanding procedure for a colorectal surgeon.

In patients who need to be operated for anal fistula, even prior to the examination, it may be clear that there is a risk of a postoperative incontinence, based on an assessment of bowel habits and obstetric history. Elderly patients, multiparous women, patients with inflammatory bowel disease (IBD) or previous proctological operations, or those with a short anal canal and weak sphincters (i.e., poor voluntary contraction) at the proctological examination are at risk for postoperative fecal incontinence (FI). We also need to investigate any previous episodes of FI.

A careful proctological examination is essential. The patient should be asked to squeeze, and the examiner should assess whether the voluntary contraction lasts at least 20–30 s. Next, a simple maneuver can be performed to elicit the anal reflex by gently touching the perianal skin with a fine needle. This may reveal signs of pudendal neuropathy. Rectal sensation may also be measured by gradually inflating a rubber balloon in the rectum. A reduced sensation and capacity of the rectal reservoir may predict postoperative fecal incontinence.

Transanal ultrasound and anorectal manometry can also help identify in advance whether the patient is at risk for p.o. FI.

Integrity of anal sphincters at anal ultrasound and a good resting tone and voluntary contraction at manometry indicate that the patient is not at risk for p.o. FI (unless the anal sphincters are grossly disrupted during surgery).

## Prevention of postoperative fecal incontinence: sphincter-sparing procedures

Let us suppose that we are dealing with a female patient with an anterior transphincteric fistula track, a short anal canal, and a lack of integrity of the distal external sphincter due to previous troublesome vaginal deliveries. If the fistula is a high trans-sphincteric type, we should be aware that performing a lay-open procedure will have a 90% chance of curing the sepsis, but the patient is likely to suffer from anal incontinence. This is because, anteriorly, the muscular bulk of the puborectalis is absent, providing less protection to the sphincter during surgical division.

Therefore, an alternative procedure is needed that preserves the external sphincter, or at least most of its length.

The simplest and cheapest way is to use a cutting seton [1].

## Cutting seton

The seton should be tied without tension around the segment of muscle to be spared. It will then gradually move downward, as the body treats it as a foreign object and attempts to expel it.

I personally divide the distal half of the internal sphincter, as I was taught at St Mark’s Hospital by Sir Alan Parks, with the aim of draining the intersphincteric plane.

Every 2 weeks, the surgeon should tie the seton distal to the sphincter, until the seton is spontaneously expelled. If necessary, a small incision of less than 1 cm will be made to remove it in the subcutaneous part of the external sphincter.

At the end of this process, the anterior part of the external sphincter remains intact, although it will have a scar resulting from the descent of the seton. This may affect the elasticity of the muscle, causing slight anal incontinence.

To achieve full continence, the patient is encouraged to perform sphincter exercises, i.e., 20 contractions a day lasting 20 s.

## Ayurvedic seton

An alternative to the silk seton is the Indian ayurvedic seton, which is covered by a number of substances that have curative properties. The most impressive study was published by Kumar and Hanifa in 2025 [2]. The authors used a slightly modified Ayurvedic seton in 215 patients with trans-sphincteric fistulas, who were followed up for 1 year. The operative time was 15 min, and the hospital stay lasted 2 days. Postoperative pain was low, with an average score of 1.16 out of 10. Only one patient experienced recurrence after 6 months, and no cases of anal incontinence were reported.

## Loose seton

The loose seton, which may be either silk or rubber, is used when the aim of the surgeon is to drain the intersphincteric plane [3], such as in a patient with Crohn’s disease who requires abscess management while receiving infliximab. The loose seton does not represent a risk for anal incontinence as it does not traumatize the anal sphincter.

## Re-routing procedures

Using advanced surgical techniques, it is possible to “reroute”—that is, “change” or “displace”—a complex fistula tract, making it surgically more accessible and reducing the risk of iatrogenic anal incontinence [4, 5].

## Anal plugs

The main advantage of anal plugs is that they do not cause postoperative incontinence. Aho Falt et al. [6] reported on 95 patients with complex anal fistulas who were preoperatively treated with a loose seton before surgery. Thirty had Crohn’s disease. A total of 151 plug procedures were performed. After a median period of 110 months, the overall healing was 38%, i.e., less than half of the patients were cured. The morbidity was low; therefore, the authors suggest that plugs can be considered a first-line treatment. This conclusion may be rather optimistic, given the high cost of the device.

## Revised lift (Phillips’ ligation of the intersphincteric fistula track) or Royakanasul procedure

The Thai surgeon modified and popularized the LIFT procedure, which involves opening the intersphincteric plane without injuring the anal sphincter, followed by ligation and division of the fistula tract at the upper part of the intersphincteric plane. Royakanasul was able to find a fistula track in most cases presenting with an acute anorectal abscess. His procedure described in 2021 [7] was to some extent “revolutionary,” as he not only drained the abscess but also explored the intersphincteric plane and eradicated sepsis in 66 of 86 cases. The wound healed in 90 days. No patient had postoperative incontinence.

## Adipose tissue (stem cells) injection

Adipose tissue injections, a rich source of mesenchymal stem cells, have been successfully used to promote anal fistula healing. Nine patients had seton removal, fistula tract excision or curettage, and a mucosa flap or a suture of the internal opening. All fistulas were high trans-sphincteric. Six cases achieved complete fistula healing at a mean follow-up of 18 months [8].

## Video-assisted ablation of the fistula tract (VAAFT)

Key steps are the visualization of the fistula tract using the fistuloscope and localization of the internal fistula opening; 87% of the patients healed after 1 year [9].

A meta-analysis was carried out by a Chinese group [10] on a total of 10 articles (779 patients). The average follow-up time was 22 months. The ratio of high to low fistulas was 6.6 to 1. The internal opening was detected in 98% of cases, which is a clearly positive finding.

The recurrence rate was less favorable, at 24% after approximately 2 years.

## Laser (FiLaC) and rectal advancement flap (RAF)

RAF is currently the gold standard but has recurrence rates between 20% and 50% and occasional soiling. The authors [11] compared this procedure with the minimally invasive laser FiLaC, using the Vaizey incontinence score and the quality-of-life scale. Based on the trial results, FiLaC could be considered a first-line treatment for high trans-sphincteric fistulas, given that the rectal advancement flap, although a sphincter-sparing procedure with high success rates, is time-consuming and more invasive.

## A comparison of four sphincter-saving techniques

Forty-nine articles with 3520 patients were included [12], reporting the results of four surgeries for anal fistulae: LIFT (intersphincteric fistula ligation), EAF (endoanal flap), FiLaC (laser), and VAAFT (video assisted), i.e.m two “hand-made” and two “instrumental.” According the meta-analysis, all these techniques may represent a valid option in the treatment of anal fistulae.

The author’s opinion is that there does not necessarily need to be a “gold standard.”

Every patient, and every abscess or fistula, may differ from another. There is no single “best” operation, only the best approach for each individual case. Therefore, an eclectic approach might be considered justified, as patients with anal sepsis exhibit a wide variety of clinical presentations, anatomical patterns, immune responses (often neglected), and symptoms.

## Data Availability

No datasets were generated or analyzed during the current study.
